# Widespread Use and Frequent Detection of Neonicotinoid Insecticides in Wetlands of Canada's Prairie Pothole Region

**DOI:** 10.1371/journal.pone.0092821

**Published:** 2014-03-26

**Authors:** Anson R. Main, John V. Headley, Kerry M. Peru, Nicole L. Michel, Allan J. Cessna, Christy A. Morrissey

**Affiliations:** 1 School of Environment and Sustainability, University of Saskatchewan, Saskatoon, Saskatchewan, Canada; 2 Aquatic Contaminants Research Division, Water Science and Technology Directorate, Environment Canada, Saskatoon, Saskatchewan, Canada; 3 Science and Technology Branch, Agriculture and Agri-Food Canada, Saskatoon, Saskatchewan, Canada; 4 Department of Biology, University of Saskatchewan, Saskatoon, Saskatchewan, Canada; Texas Tech University, United States of America

## Abstract

Neonicotinoids currently dominate the insecticide market as seed treatments on Canada's major Prairie crops (e.g., canola). The potential impact to ecologically significant wetlands in this dominantly agro-environment has largely been overlooked while the distribution of use, incidence and level of contamination remains unreported. We modelled the spatial distribution of neonicotinoid use across the three Prairie Provinces in combination with temporal assessments of water and sediment concentrations in wetlands to measure four active ingredients (clothianidin, thiamethoxam, imidacloprid and acetamiprid). From 2009 to 2012, neonicotinoid use was increasing; by 2012, applications covered an estimated ∼11 million hectares (44% of Prairie cropland) with >216,000 kg of active ingredients. Thiamethoxam, followed by clothianidin, were the dominant seed treatments by mass and area. Areas of high neonicotinoid use were identified as high density canola or soybean production. Water sampled four times from 136 wetlands (spring, summer, fall 2012 and spring 2013) across four rural municipalities in Saskatchewan similarly revealed clothianidin and thiamethoxam in the majority of samples. In spring 2012 prior to seeding, 36% of wetlands contained at least one neonicotinoid. Detections increased to 62% in summer 2012, declined to 16% in fall, and increased to 91% the following spring 2013 after ice-off. Peak concentrations were recorded during summer 2012 for both thiamethoxam (range: <LOQ - 1490 ng/L, canola) and clothianidin (range: <LOQ – 3110 ng/L, canola). Sediment samples collected during the same period rarely (6%) contained neonicotinoid concentrations (which did not exceed 20 ng/L). Wetlands situated in barley, canola and oat fields consistently contained higher mean concentrations of neonicotinoids than in grasslands, but no individual crop singularly influenced overall detections or concentrations. Distribution maps indicate neonicotinoid use is increasing and becoming more widespread with concerns for environmental loading, while frequently detected neonicotinoid concentrations in Prairie wetlands suggest high persistence and transport into wetlands.

## Introduction

Degradation of aquatic ecosystems from chemical inputs is a global concern because of the loss of ecosystem services provided through water supplies, food resources and habitat for species of fish and wildlife. Wetlands are some of the most sensitive, biologically diverse, and globally productive ecosystems [1]. Worldwide, the rate of loss and deterioration of wetlands is accelerating due to increasing anthropogenic impacts affecting their overall ecological condition [2]. Wetlands in agricultural areas in Canada are under serious threat from expanding agricultural intensification; specifically, increased reliance on chemical fertilizers and pesticides (herbicides, fungicides, and insecticides). There is a growing concern that these inputs are degrading wetland water quality and, consequently, impacting aquatic and wetland-dependent terrestrial species. With over 50% of the wetlands in the Prairie Pothole Region (PPR) of Canada historically drained, the remaining intact wetlands are under stress due to eutrophication, sedimentation, loss of vegetation and tillage of marginal lands as a result of agricultural activity [3]. Farming has shifted toward large-scale production, mechanization and mono-cropping. Researchers estimate an exponential growth in chemical inputs designed for improved agricultural yields – specifically, the increased use of insecticides [4].

Current agricultural practices are dependent on a newer class of insecticides, the neonicotinoids. Valued for their versatility in application [5]–[7] and widely used throughout Europe and North America, these chemicals represent the fastest growing class of insecticides globally since the introduction of the pyrethroids. The extensive use of the neonicotinoids is largely due to their effectiveness and broad spectrum toxicity to a wide range of pests [8]. Eighty percent of all treated seeds are coated with a neonicotinoid insecticide [5]. Seeds of the major Prairie crops in Canada (e.g., canola, wheat, barley, oat and field pea) are commonly coated with one of the neonicotinoid active ingredients clothianidin, imidacloprid, or thiamethoxam while acetamiprid is also used on fruit or leafy vegetable crops. The Canadian Prairie Pothole Region (PPR) consists of 39 million hectares (ha) and accounts for 98% of the country's canola production – over 8.5 million ha were seeded in 2012 [9] of which nearly all were seeded with neonicotinoid-treated seed (PMRA pers. comm).

Neonicotinoids - systemic insecticides - contain an active ingredient that translocates throughout the growing plant and acts on the nervous system of insect pests [10]. Recent concern over this class of insecticides is, in part, due to their acute toxicity to non-target insects such as bees and aquatic invertebrates [11]–[16]. In addition, some of the neonicotinoids have relatively long half-lives in soil (e.g., thiamethoxam DT_50_ = avg. 229 days; clothianidin DT_50_ = 148–1,155 days) and high water solubility (e.g., thiamethoxam  = 4,100 mg/L; clothianidin  = 327 mg/L) [17] leading to environmental persistence and high potential for transport into surface waters via surface runoff or groundwater discharge [18]–[19].

From 1971 to 1991, pesticide use in Canada increased by 500% resulting in a greater quantity of pesticides susceptible to transport [20]. Today, more pesticides are used in the Prairies than any other region of Canada. Wetlands in the PPR typically occupy topographic depressions that naturally accumulate surface runoff which may contain pesticides from adjacent/surrounding agricultural fields [20]. Millions of PPR wetlands drain surrounding agricultural fields and accumulate snowmelt and (to a lesser extent) summer rainfall [21]–[22] potentially making them susceptible to neonicotinoid contamination. For example, up to 24% of Saskatchewan's wetlands may surpass pesticide regulatory requirements for protection of aquatic life during storm events [23]. During high rainfall events, Prairie wetlands in flooded agricultural landscapes were found to contain an average of 19 herbicides and insecticides [24].

Many western nations are examining the distribution and use of neonicotinoids along with impacts on ecosystem health [18]. However, the actual distribution and concentration of neonicotinoids in North American surface water systems remains poorly known with the exception of limited published studies focused on imidacloprid in rivers and streams [19], [25]–[26] and one study reporting thiamethoxam and acetamiprid detections in playa wetlands of Texas [27]. In the PPR agricultural-wetland landscape, the actual distribution of use of neonicotinoids and their levels in agricultural wetlands remains unknown. Therefore, our objectives were to: 1) develop geospatial maps of current neonicotinoid use within the PPR in relation to annual crop plantations and 2) survey levels of neonicotinoids in water and sediment of a subset of wetlands surrounded by different crops (grasslands, barley, canola, oat, wheat and field pea) through time. We hypothesized that neonicotinoid applications would be highest in areas of intensive canola production and neonicotinoid concentrations and detections in wetlands would similarly be higher in canola fields, particularly during the summer growing season.

## Methods

### Ethics Statement

No ethics approval was required. We obtained right-of-entry agreements for water sampling and sediment collection on community pastures (RM of Wolverine - Agriculture and Agri-Food Canada), Ducks Unlimited lands (Ducks Unlimited Canada) and St. Denis National Wildlife Area (Environment Canada) in addition to private landowner permission. Private landowners who granted access in this study wish to remain anonymous and specific GPS coordinates cannot be provided as part of that confidentiality. Our field studies did not involve endangered or protected species.

### Study Area for Wetland Sampling

Our study was carried out across a 32-km^2^ area in central-east Saskatchewan. Water and sediment samples were collected from wetlands situated in agricultural fields near the communities of St. Denis (52° 10′22″ N, 106° 5′57″ W), Colonsay (51° 59′ 0″ N, 105° 53′ 0″ W), Lanigan (51° 51′ 0″ N, 105° 2′ 0″ W) and Humboldt (52° 12′ 7″ N, 105° 7′ 23″ W). The study fields were selected to represent the range of Prairie crop types located in zones of intensive agricultural production and neonicotinoid use as well as a high density of pothole wetlands.

### GIS Mapping of Neonicotinoid Applications

Pesticide use reporting in Canada is currently considered confidential and seed treatments are poorly monitored. To estimate the spatial distribution of neonicotinoid use across the Canadian Prairies, we integrated standard pesticide application rate recommendations for registered uses of seed treatment products and their associated crops (Saskatchewan Ministry of Agriculture, 2011) [28], percentage of each crop treated with neonicotinoids (2009–2010 confidential PMRA data), and remote-sensing field-level crop inventory maps (Agriculture and Agri-Food Canada) into a geographic information system (GIS; ArcMap 10, Environmental Systems Resource Institute, Redlands, CA). Data on PPR cropland distribution was derived from Agriculture Canada's remotely sensed land cover maps at 56-m resolution (2009–2010) and 30-m resolution (2011–2012). For our analysis, cropland of interest included all land potentially planted with treated seed including: barley, canola, corn, dry bean, field pea, mustard, oat, soybean and wheat. Percentages of singular treated crops were then extracted from remote sensing crop maps based on available 2010 confidential PMRA data to isolate treated land areas from total planted areas. Integrated maps were individually created for three neonicotinoid compounds (thiamethoxam, clothianidin, imidacloprid) and year (2009–2012). Because we were primarily interested in seed treatments of grain crops, acetamiprid maps were not compiled because Prairie crop-use data were limited to potato which is treated both with a seed treatment and foliar spray. We determined the neonicotinoid application rate via treated seed by multiplying grams of neonicotinoid active ingredient (AI) per kilogram of seed by the seeding rate of kilograms seed per hectare. This produced a rate of grams of active ingredient per hectare (g AI/ha). For crops potentially using more than one application rate, we conservatively used median recommended guidelines (e.g., thiamethoxam: barley  = 13 g/ha, canola  = 21 g/ha, beans  = 26 g/ha). We calculated the pixel equivalent of a hectare for all raster maps by dividing raster resolution by size of an actual hectare (e.g., resolution  = 56 m×56 m/ha = 100 m×100 m). We then used a conditional statement in ArcMap Spatial Analyst tools to multiply the hectare equivalent by calculated application rate (g AI per specific crop) to determine an estimated value for each hectare planted to one crop type. Because the majority of field crops are planted on a quarter section level (65 ha), all individual crop maps by AI were merged together (by specific year) and summed to estimate total neonicotinoid distribution throughout the PPR.

### Water Sampling

We used the Dominion Land Survey system [29] which divides agricultural land across the Canadian Prairies into 1.6-km^2^ sections (260 ha) containing four quarter sections (65 ha) to delineate zones for wetland sampling because crops are planted at the quarter section scale. We sampled, where available, three replicate wetlands from each of 50 quarter sections across a range of wetland classes (Class II: temporary ponds; Class III: seasonal ponds; Class IV: semi-permanent ponds; and Class V: permanent ponds). Fields were randomly selected to represent five agricultural crop types in the study area (canola, barley, wheat, oat and field pea) in addition to grasslands/hayfields. In total, water samples from 136 wetlands in 50 quarter sections were collected for analysis; 89% of wetlands sampled were situated in crop fields as follows: canola (40%), barley (20%), wheat (18%), oat (11%), field pea (0%) with 11% of the wetlands situated in grassland and hayfield (reference) areas. In spring 2012, there were no wetlands situated in fields seeded to field pea the previous year, but wetlands in pea fields were sampled in subsequent water collections. Water samples from the same wetlands were collected four times: between snowmelt and seeding in spring 2012 (April), during the growing season in summer 2012 (June), after harvest in fall 2012 (September) and again between snowmelt and seeding in spring 2013 (May). Collection sites were accessible by foot and samples were collected centrally in each wetland beyond surrounding edge vegetation and, where possible, distant from submerged aquatic vegetation. One litre (L) of water was collected using a subsurface grab at a depth of 10 cm in chemically cleaned (acetone: hexane washed) amber glass jars. Bottles were sealed with Teflon-lined caps and then stored in the dark during transport and refrigerated at 4°C until analysis.

The type of crop surrounding each wetland was determined from landowner crop rotation schedules or by plant identification. GPS coordinates and photographs of each study wetland were recorded to ensure the same wetlands were sampled in subsequent sampling periods.

### Sediment Sampling

During the summer 2012 water collection, we also collected sediment cores from each study wetland. Sediment sampling involved collection of three replicate 0- to 6-cm depth samples from three zones within each wetland: centrally, the zone of emergent vegetation and that of submerged vegetation. Sediment was collected using a 1.2-m black PVC pipe with a 15-cm diameter opening and 0.6-cm holes drilled into the lower sides of the pipe to allow water to evacuate during coring. The combined sediment cores were pooled to yield a sediment sample of approximately 1 kg. Sediment was placed in polyethylene freezer bags, transported to the laboratory in a large cooler and then immediately placed in a freezer at −20°C until analysis.

### Chemical Analysis

Wetland water and sediment samples were analyzed at the National Hydrology Research Centre, Environment Canada, Saskatoon, SK using methods adapted from that of Xie et al. [30]. Analytical standards of thiamethoxam, clothianidin, imidacloprid and acetamiprid were from Chem Service (West Chester, PA, USA) and the internal standard, d_4_-imidacloprid, from CDN Isotopes (Pointe-Claire, Quebec, CA).

#### Sample Extraction

In brief, water samples (500 mL) were passed through Oasis HLB cartridges (Waters, Mississauga, Canada) which had been sequentially conditioned with methanol (10 mL) and water (10 mL). After sample loading, the cartridges were washed with de-ionized water (5 mL) to remove salts and the cartridges were dried under vacuum for 5 min. The retained analytes were eluted with methanol (10 mL), the eluates were evaporated to dryness and the extract residues reconstituted in 500 μL of water followed by addition of the internal standard. Sediment samples (5.0 g wet-weight) and acetonitrile (10 mL) were sonicated (30 min) and then centrifuged (15 min @ 5000 rpm) and the supernatant decanted. Following a second sonication and centrifugation, the combined decantates were evaporated to ∼1 mL, taken to a final volume of 2 mL with water, and internal standard added.

#### LC/MS/MS Analysis

A Waters 2695 Alliance HPLC system (Waters Corp., Milford, MA), consisting of a solvent degassing unit, pump and autosampler, was used with a Waters XTerra MS-C_8_ (3.5-μm dia. particle size) column (2.1-×100-mm) (Waters Corp., Milford, MA) at 30°C. Isocratic elution of the analytes was achieved with an 80/20 mix of solvent A (100% water and 0.1% formic acid) and solvent B (90% acetonitrile, 10% water and 0.1% formic acid). The run time was 10 min and the injection volume was 20 μL.

The neonicotinoid insecticides were quantitated (internal standard method) and their presence confirmed using the Micromass Quattro Premier triple quadrupole mass spectrometer (Waters Corp., Milford, MA) equipped with an electrospray ionization interface set to positive ion mode. Ionization and MS/MS conditions were optimized by infusing a 0.5 mg/L solution of each insecticide into the ion source in a 50∶50 (v/v) acetonitrile:water solution with a syringe pump. MRM transitions, selected from the product ion scan and optimal cone voltages and collision energies for each neonicotinoid are provided in Table S1. Other instrumental settings were as follows: source temperature, 90°C; capillary voltage, 3.00 kV; extractor voltage, 5.00 V; desolvation temperature, 240°C; nitrogen desolvation gas flow rate, 476 L/h; nitrogen cone gas flow rate, 38 L/h; nitrogen nebulizer gas flow rate was at maximum flow; multiplier voltage, 657 V; and the interchannel delay was 0.10 s. Argon was used as the collision gas at a pressure which increased the Pirani gauge reading to 3.12×10^−4^ mbar. Resolution was set to achieve unit mass resolution for quadrupole 1 and approximately 2 amu resolution for quadrupole 3.

A four-level calibration curve (5 to 50 μg) was analyzed before and after each batch of 10 samples which also contained a laboratory or field blank and a fortified sample. Limits of quantification (LOQ) in water were as follows: thiamethoxam, 1.8 ng/L; clothianidin, 1.2 ng/L; imidacloprid 1.1 ng/L; and acetamiprid, 0.5 ng/L. Mean recoveries (n = 33) from Milli Q (n = 8) and river (n = 3) water each fortified at 500, 100 and 50 ng/L were as follows: thiamethoxam, 88.8±3.4%; clothianidin, 78.9±5.4% (mean ± SD); imidacloprid, 85.9±3.9% and acetamiprid, 89.6±3.7%. Mean recoveries from sediment fortified at 20 μg/kg (n = 5) were as follows: thiamethoxam: 73.6±5.2%; clothianidin: 72.3±7.0%; imidacloprid: 73.5±7.1%; and acetamiprid: 74.5±5.9%. All neonicotinoid concentrations were corrected for recovery and all laboratory and field blanks were below detection.

### Statistical Analysis

Given the structural similarity of clothianidin, thiamethoxam, imidacloprid and acetamiprid and their cumulative and irreversible binding to insect nicotinic acetylcholine receptors, individual neonicotinoids are assumed to be additive in relative toxicity [31]. Therefore, concentrations of multiple neonicotinoids detected in a given sample were summed and presented as total neonicotinoids.

We used a general linear mixed model (GLMM) in package lme4 in R (R Core Team 2013) to investigate the effects of crop type (grassland, barley, canola, field pea, oat, wheat) on changes in wetland total neonicotinoid concentration over one full agricultural growing season (April 2012 to May 2013). A GLMM with a Gaussian distribution was used because total neonicotinoid concentration met the assumption of normally-distributed residuals after log transformation. Crop type and time were fixed effects; wetlands nested within quarter sections and the slope of change in neonicotinoid concentrations over time were random effects; and baseline (spring 2012) neonicotinoid concentration and prior year's (2011) crop type were covariates. We had an unbalanced design because some ponds could not be resampled due to wetland drawdown (fall 2012) and spring 2013 overflooding. We used Akaike's information criteria (AIC) to identify the best distribution and to decide whether to retain slope and intercept random effects [32]. Significant interactions of crop type and time were examined using *post-hoc* testing of interaction contrasts in package “phia” [33]. We corrected for multiple comparisons and associated Type I errors using the Dunn-Šidák correction, because it has more power than Bonferroni [34].

## Results

### Neonicotinoid distribution in the Prairies

**Figure 1:**
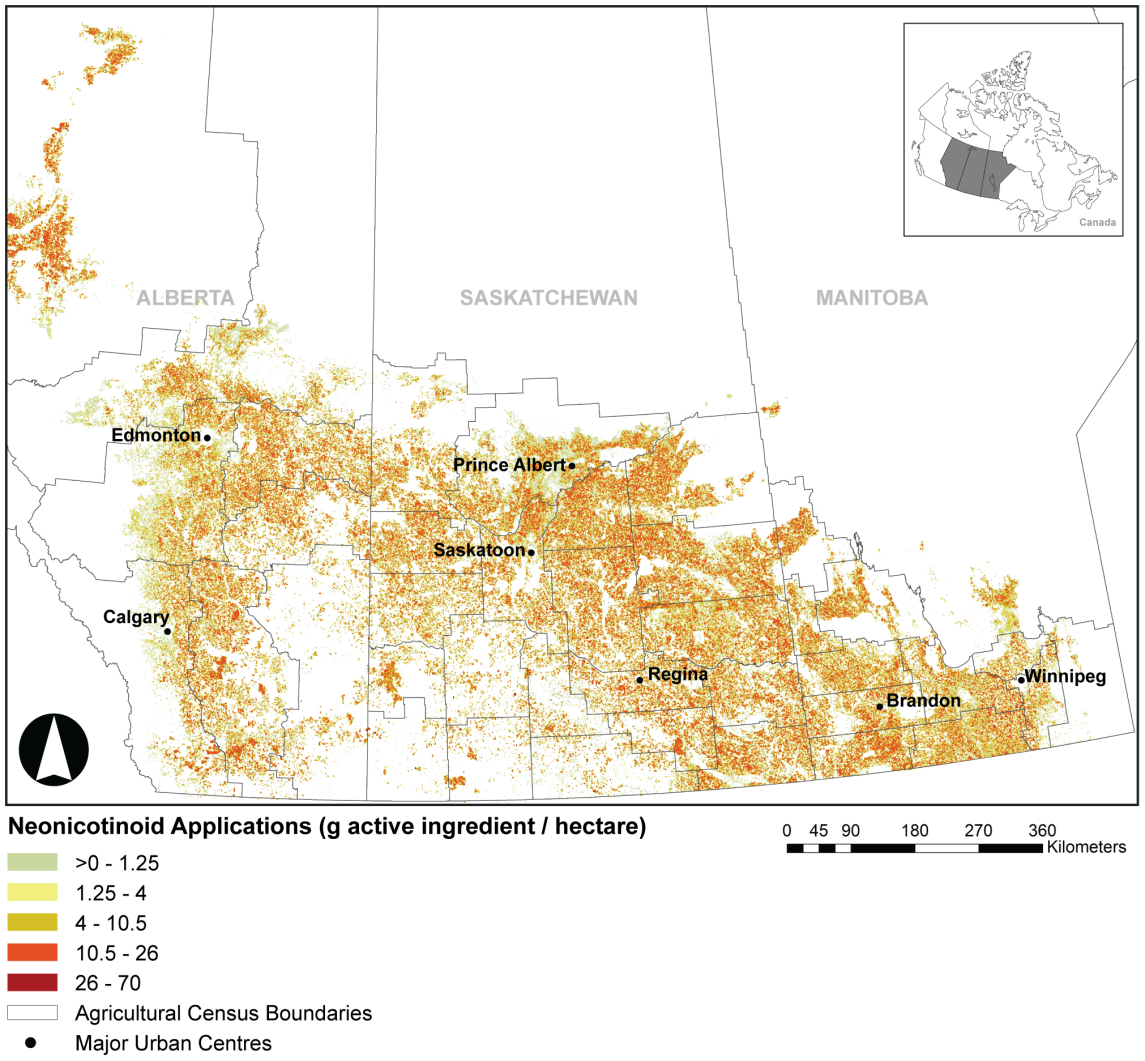
Map of modelled distribution of neonicotinoid use across Prairie Canada: Alberta, Saskatchewan and Manitoba (2012). Neonicotinoid application rates (g AI/ha) represent the sum total of clothianidin, imidacloprid and thiamethoxam across an agricultural quarter section (65-ha field) on all crops predicted to be treated with neonicotinoid seed treatments. Acetamiprid is not included because Prairie crop-use data were limited to potato which is treated both with a seed treatment and foliar spray.

**Figure 2:**
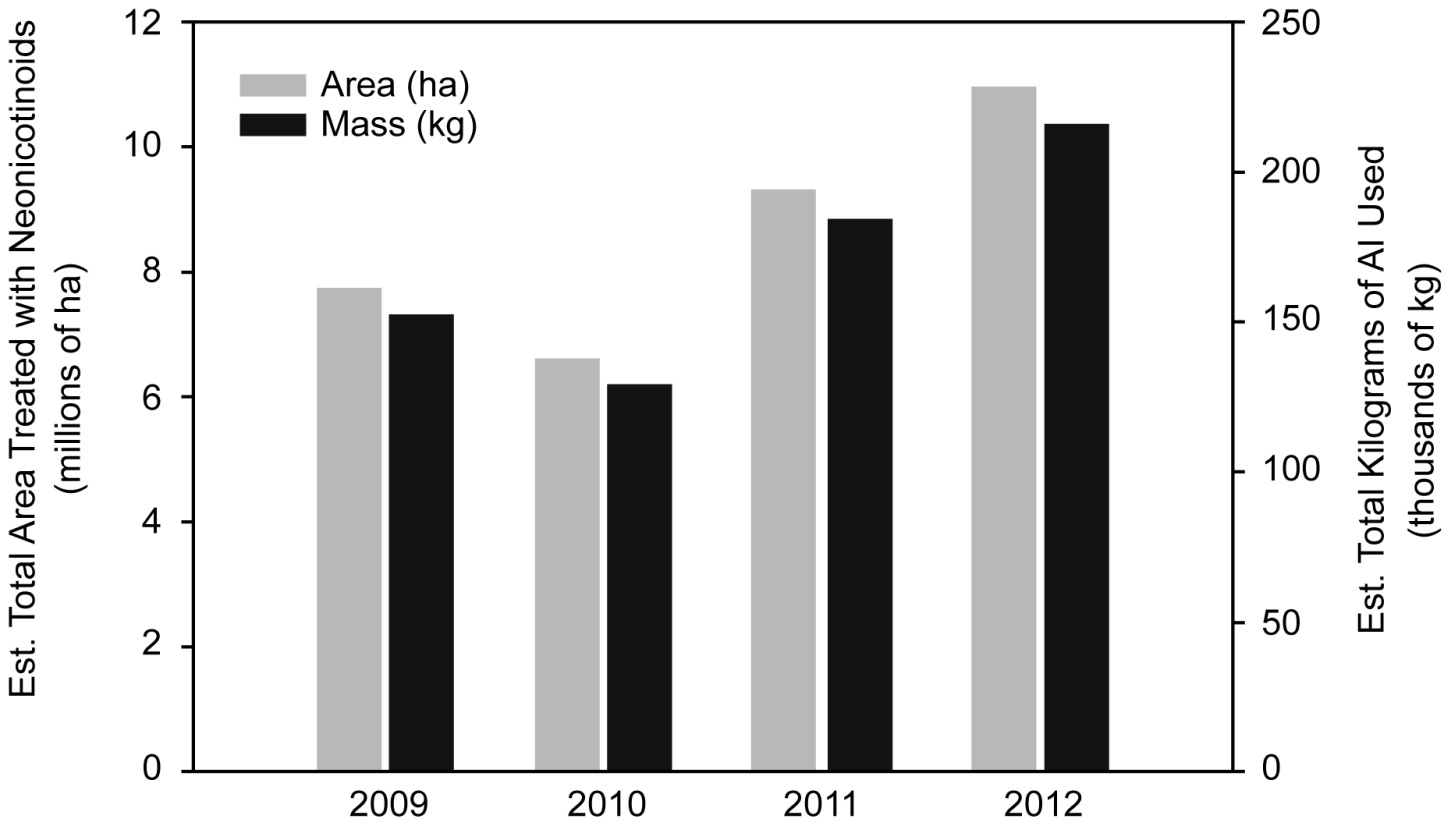
Estimated total neonicotinoid distribution across Prairie Canada. Area of total agricultural land (millions of ha) using a neonicotinoid seed treatment and estimated total mass (kg) of active ingredient (AI) applied across the Canadian Prairie region from 2009 to 2012. Composite area and mass values include all predicted treated crop types and neonicotinoid active ingredients (clothianidin, imidacloprid and thiamethoxam) based on extrapolation of mapped distribution.

Our predictive maps indicated broad neonicotinoid distribution and application rates across the Canadian Prairies (range: >0–70 g/ha) (Fig. 1; Fig. S1–S3) with further GIS analysis showing a trend of increasing use over time. By 2012, nearly 11 million hectares (est. total Prairie cropland  = 25 million ha) of cropland across the Canadian Prairies was estimated to be treated with clothianidin, thiamethoxam and imidacloprid; an approximate 30% increase from 2009 (7.7 million ha; Table 1, Fig. 2). Most treated areas fell in the medium range of application rates (4–10.5 g/ha). We conservatively estimate that total combined mass of neonicotinoids used across Alberta, Saskatchewan and Manitoba ranged between 129,000 kg (2010) to 216,000 kg (2012; Fig. 2). This also represents a significant proportion of the total annually seeded cropland in the Prairies ranging from 31% in 2009 to 44% in 2012 (Table 1). Remote sensing data of cropland in Manitoba was not completed by Agriculture Canada in 2010, and therefore not included, which may explain the decrease in estimated neonicotinoid use. The increasing trend is evident in spite of the wet springs of 2010 and 2011, when a substantial area (2.9 million ha in 2010 and 3.1 million ha in 2011) of cropland was not seeded (Table 1).

**Table 1:** Estimated distribution of cropland area treated with neonicotinoids in the Prairie Pothole Region (PPR) of Canada (2009–2012).

Year^2^	Application Category	Application Rate (g AI/ha)	% Within Category	Est. Area Treated (millions ha)
	Low	>0–1.25	23.2	2.55
	Low-Medium	1.25–4	20.6	2.27
**2012**	Medium	4–10.5	30.2	3.32
	Medium-High	10.5–26	25.8	2.84
	High	26–70	<1	0.013
				**10.9 (44%)**
	Low	>0–1.25	21.9	2.05
	Low-Medium	1.25–4	22.7	2.12
**2011**	Medium	4–10.5	31.6	2.96
	Medium-High	10.5–26	23.8	2.23
	High	26–70	<1	0.006
				**9.37 (42%)**
	Low	>0–1.25	15.8	1.05
	Low-Medium	1.25–4	23.5	1.55
**2010**	Medium	4–10.5	37.1	2.45
	Medium-High	10.5–26	23.5	1.55
	High	26–70	<1	0.007
				**6.61 (30%)**
	Low	>0–1.25	17.5	1.36
	Low-Medium	1.25–4	24.6	1.90
**2009**	Medium	4–10.5	37.0	2.87
	Medium-High	10.5–26	20.7	1.61
	High	26–70	<1	0.008
				**7.75 (31%)**

Area and percentage of seed treatment applications are separated into 5 categories defined by application rates. Bolded values are the total area treated and percentage of Prairie croplands^1^.

1 Total PPR cropland in production based on Statistics Canada Field Crop Reporting Series: *July 2009, July 2010, July 2011, July 2012: Estimates of Principal Field Crops.*

2 In both 2010 (Est. 2.9 million ha) and 2011 (Est. 3.1 million ha), wet spring conditions increased the amount of cropland that went unseeded.

Neonicotinoid treated areas and application rates (g/ha) differ by crop and active ingredient. In 2009, the dominant crops (by area) with neonicotinoid seed treatments ranked as follows: canola > wheat > corn > field pea > barley > oat. By 2012, that ranking had changed slightly to canola > wheat > soybean > corn > barley > field pea > dry bean > oat. Although canola and wheat seed treatments cover the largest area, field pea treated with thiamethoxam was calculated to have the highest application rate (70 g/ha) while oat had the lowest calculated application rate (12 g/ha). Thiamethoxam (5.8 million ha) covered the broadest spatial extent due to the range of crops on which it is currently used as a seed treatment (e.g., canola, wheat, barley). Clothianidin (5.1 million ha) was the second most widely used neonicotinoid whereas imidacloprid (45,000 ha) was substantially less. The application area for acetamiprid was not calculated because Prairie crop-use data were limited to potato which is treated both with a seed treatment and foliar spray. Overall, maximum neonicotinoid use occurred in regions with intensive canola (Peace River region of Alberta, central Saskatchewan and southwestern Manitoba) and soybean production (southeastern Manitoba). Our results suggest that the neonicotinoids are widely used in the Canadian Prairies and that PPR wetlands are generally surrounded by crops treated with neonicotinoids which likely increases their risk of contamination with neonicotinoid insecticides.

### Neonicotinoid concentrations in water

In spring 2012, between snowmelt and seeding, 36% of (49/136) wetlands sampled contained at least one neonicotinoid. By summer 2012, the number of wetlands with detectable concentrations of neonicotinoids had increased to 62% (83/134) after seeding (Table 2). After harvest (fall 2012), 16% (13/80) wetlands contained trace neonicotinoid concentrations. Of the wetlands that were accessible for re-sampling the following spring (2013), 91% (82/90) had detectable neonicotinoid concentrations. At the field level, neonicotinoids were detected in wetlands on 29 of 52 quarter sections in spring 2012 (56%); 37 of 49 quarter sections in summer 2012 (76%); 11 of 38 quarter sections in fall 2012 (29%) and 33 of 35 quarter sections in spring 2013 (94%). Detections of neonicotinoids in wetlands included all crop types and grassland samples.

**Table 2:** Summary of detections, arithmetic means and maximum concentrations of total neonicotinoids and active ingredients in water from Prairie wetlands of central Saskatchewan (2012–2013).

Season				Total Neonic. (ng/L)^1^		Imidacloprid (ng/L)		Thiamethoxam (ng/L)		Clothianidin (ng/L)		Acetamiprid (ng/L)	
**Spring 2012 (pre-seeding)**	**Crop**	**Wetlands (n)**	**Detection (%)**	**Mean**	**Max**	**Mean**	**Max**	**Mean**	**Max**	**Mean**	**Max**	**Mean**	**Max**
	Barley	28	29	5.8	41.1	ND	ND	ND	ND	3.9	39.4	0.4	5.2
	Canola	54	52	20.7	184	1.7	30.3	2.5	19.1	16.3	144	ND	ND
	Oats	15	47	5.8	21.7	ND	ND	1.3	7.0	3.6	20.0	0.4	1.8
	Peas	0	NS	NS	NS	NS	NS	NS	NS	NS	NS	NS	NS
	Wheat	24	25	8.3	52.7	ND	ND	4.3	32.4	3.1	20.2	ND	ND
	Grassland	15	7	1.1	7.9	ND	ND	ND	ND	1.1	7.9	ND	ND
**Overall**		**136**	**36**	**8.3**	**184**		**30.3 (2%)**		**32.4 (10%)**		**144 (36%)**		**5.2 (1%)**
**Summer 2012 (growing)**	**Crop**	**Wetlands (n)**	**Detection (%)**	**Mean**	**Max**	**Mean**	**Max**	**Mean**	**Max**	**Mean**	**Max**	**Mean**	**Max**
	Barley	18	83	78.9	322	1.5	18.3	19.3	91.3	57.8	277	ND	ND
	Canola	61	70	185	3110	1.8	67.9	40.3	1490	142	3110	1.1	54.4
	Oats	3	100	131	235	ND	ND	121	234	9.4	27.0	ND	ND
	Peas	8	50	9.6	28.4	ND	ND	ND	ND	9.6	28.4	ND	ND
	Wheat	29	62	53.5	524	15.9	256	2.3	37.7	35.0	518	ND	ND
	Grassland	15	13	2.7	5.8	ND	ND	ND	ND	0.8	4.1	0.4	2.3
**Overall**		**134**	**62**	**76.8**	**3110**		**256 (8%)**		**1490 (19%)**		**3110 (51%)**		**54.4 (1%)**
**Fall 2012 (harvest)**	**Crop**	**Wetlands (n)**	**Detection (%)**	**Mean**	**Max**	**Mean**	**Max**	**Mean**	**Max**	**Mean**	**Max**	**Mean**	**Max**
	Barley	13	8	1.1	7.0	ND	ND	ND	ND	1.1	7.0	ND	ND
	Canola	35	20	5.4	32.6	ND	ND	2.2	20.0	2.0	30.9	0.6	11.8
	Oats	3	33	4.2	12.0	ND	ND	ND	ND	ND	ND	4.2	12.0
	Peas	5	40	5.3	16.0	ND	ND	3.6	14.6	ND	ND	0.5	1.6
	Wheat	15	0	ND	ND	ND	ND	ND	ND	ND	ND	ND	ND
	Grassland	9	22	13.5	101	ND	ND	11.9	100	ND	ND	0.4	2.0
**Overall**		**80**	**16**	**4.0**	**101**		**ND (0%)**		**100 (6%)**		**30.9 (5%)**		**12.0 (5%)**
**Spring 2013 (pre-seeding)**	**Crop**	**Wetlands (n)**	**Detection (%)**	**Mean**	**Max**	**Mean**	**Max**	**Mean**	**Max**	**Mean**	**Max**	**Mean**	**Max**
	Barley	16	94	74.9	212	ND	ND	19.8	107	53.2	157	ND	ND
	Canola	51	98	53.1	178	1.4	4.8	12.6	93.5	38.5	173	ND	ND
	Oats	3	100	60.7	102	ND	ND	41.9	79.4	16.9	20.4	ND	ND
	Peas	6	100	33.3	60.6	ND	ND	ND	ND	33.3	60.6	ND	ND
	Wheat	9	89	41.4	85.3	ND	ND	18.2	58.2	21.4	30.7	ND	ND
	Grassland	5	0	ND	ND	ND	ND	ND	ND	ND	ND	ND	ND
**Overall**		**90**	**91**	**52.7**	**212**		**4.8 (2%)**		**107 (23%)**		**173 (87%)**		**ND (0%)**

1 Total neonicotinoid concentrations are the sum of all four active ingredients detected in wetland samples.

NS: not sampled; wetlands for this crop were dry, absent or overflooded.

ND: not detected; LOQ: Acetamiprid (0.25 ng/L); Clothianidin (0.6 ng/L); Thiamethoxam (0.9 ng/L); Imidacloprid (0.55 ng/L).

Thiamethoxam and clothianidin were detected in all 4 sampling periods; imidacloprid was not detected in fall 2012 and acetamiprid was not detected in spring 2013. Clothianidin was the most commonly detected neonicotinoid in water samples, and had the highest maximum and mean concentrations during three of the sampling periods: spring 2012 (max: 144 ng/L; mean: 16), summer 2012 (max: 3110 ng/L; mean: 142), and spring 2013 (max: 173 ng/L; mean: 39) (Table 2). In the fall, thiamethoxam had the highest maximum concentration (max: 100 ng/L; mean: 12).

**Figure 3:**
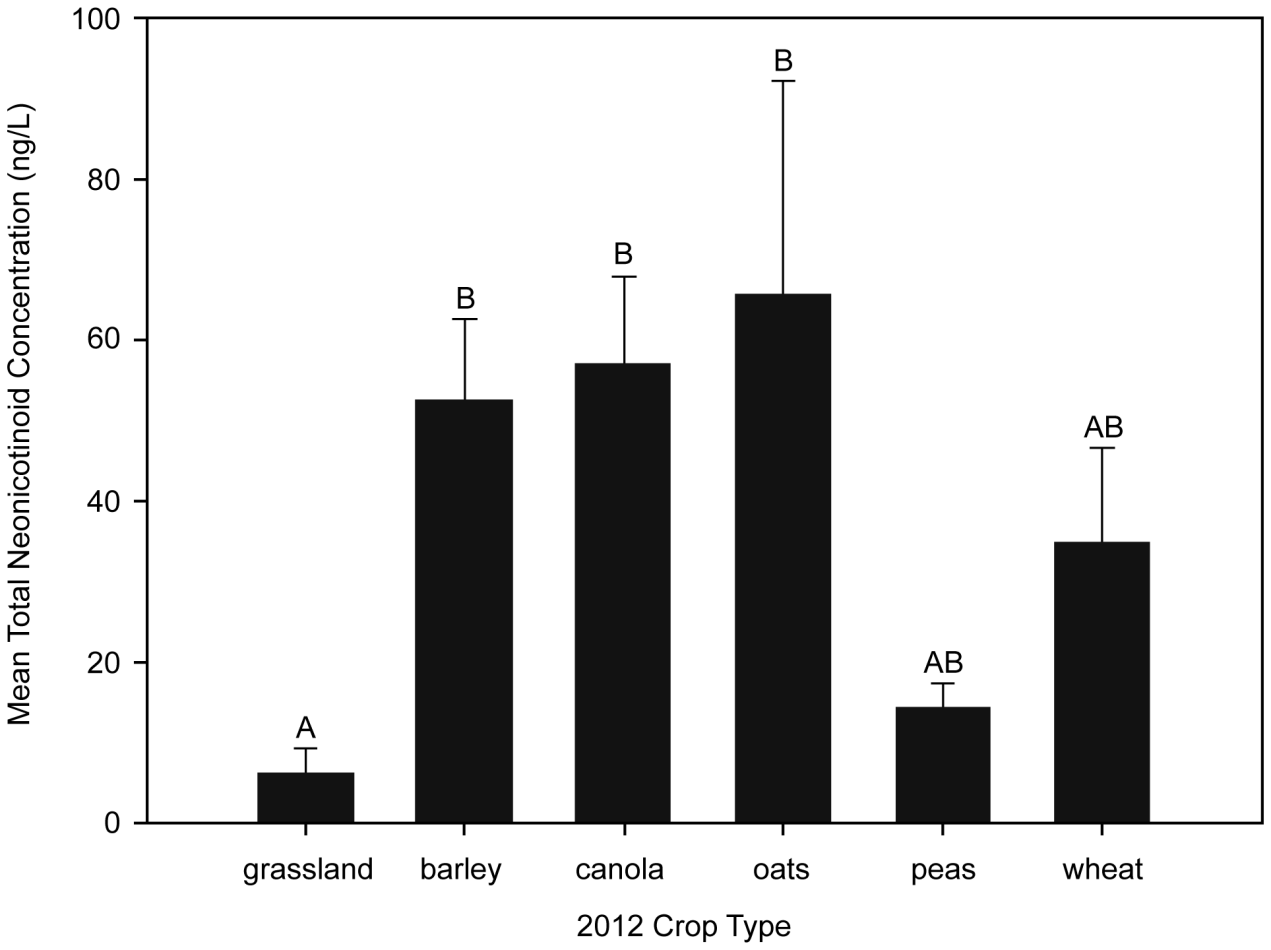
Mean total neonicotinoid water concentrations by crop type measured in wetlands in central Saskatchewan. Bars represent means (±SE) for each crop over all sampling periods in 2012–2013. Statistical comparisons (letters) of individual crops are relative to grasslands. Bars sharing the same letter (i.e. A, B) indicate no statistical difference in means.

Differences in mean concentrations between field crop types were apparent. Wetlands situated in barley, canola and oat fields had significantly higher mean annual concentrations than those in grasslands (Table 3, Fig. 3). Pre-seeding (spring 2012) concentrations had a small (β± S.E.: 0.15±0.06, *P* = 0.01), but positive effect on summer 2012 concentrations whereas previous year's (2011) crop type did not (Table 3).

**Table 3:** Results of generalized linear mixed model analyzing total neonicotinoid concentration in response to crop type and season.

Fixed Effects	β Estimate ± SE	*t*	*P*
(Intercept)	0.83±0.54	1.53	0.13
**Season** (reference: summer 2012)			
Fall 2012	0.34±0.57	0.60	0.55
Spring 2013	−0.12±0.60	−0.21	0.84
**Crop** (reference: Grassland)			
Barley	2.29±0.80	2.84	**0.007**
Canola	2.23±0.74	3.03	**0.004**
Oats	3.43±1.42	2.42	**0.02**
Peas	0.85±1.00	0.84	0.41
Wheat	1.07±0.73	1.47	0.15
**Spring 2012 Concentration**	0.15±0.06	2.51	**0.014**
**2011 Crop** (reference: Grassland)			
Barley	−0.05±0.49	−0.10	0.92
Canola	0.11±0.40	0.29	0.77
Oats	0.34±0.53	0.63	0.53
Wheat	0.22±0.49	0.45	0.65
**Season x Crop** (reference: Summer 2012 Grassland)			
Barley Fall 2012	−3.04±0.76	−3.98	**0.0001**
Barley Spring 2013	0.29±0.75	0.39	0.70
Canola Fall 2012	−2.55±0.64	−3.97	**0.001**
Canola Spring 2013	0.25±0.66	0.38	0.71
Oats Fall 2012	−3.59±1.39	−2.58	**0.011**
Oats Spring 2013	−0.82±1.29	−0.64	0.53
Peas Fall 2012	−0.96±0.97	−1.00	0.32
Peas Spring 2013	1.41±0.95	1.49	0.14
Wheat Fall 2012	−1.74±0.70	−2.48	**0.014**
Wheat Spring 2013	1.30±0.74	1.76	0.08
**Random Effects**	**Variance**		**SD**
**Season x Quarter Section**			
Summer 2012	1.14		1.07
Fall 2012	0.13		0.11
Spring 2013	0.23		0.48
**Season x Wetland** (nested within Site)			
Summer 2012	1.36		1.16
Fall 2012	0.50		0.70
Spring 2013	0.52		0.72

Total neonicotinoid concentration was measured repeatedly in up to 136 wetlands situated on 50 agricultural quarter sections in Saskatchewan during spring 2012 through spring 2013.

**Figure 4:**
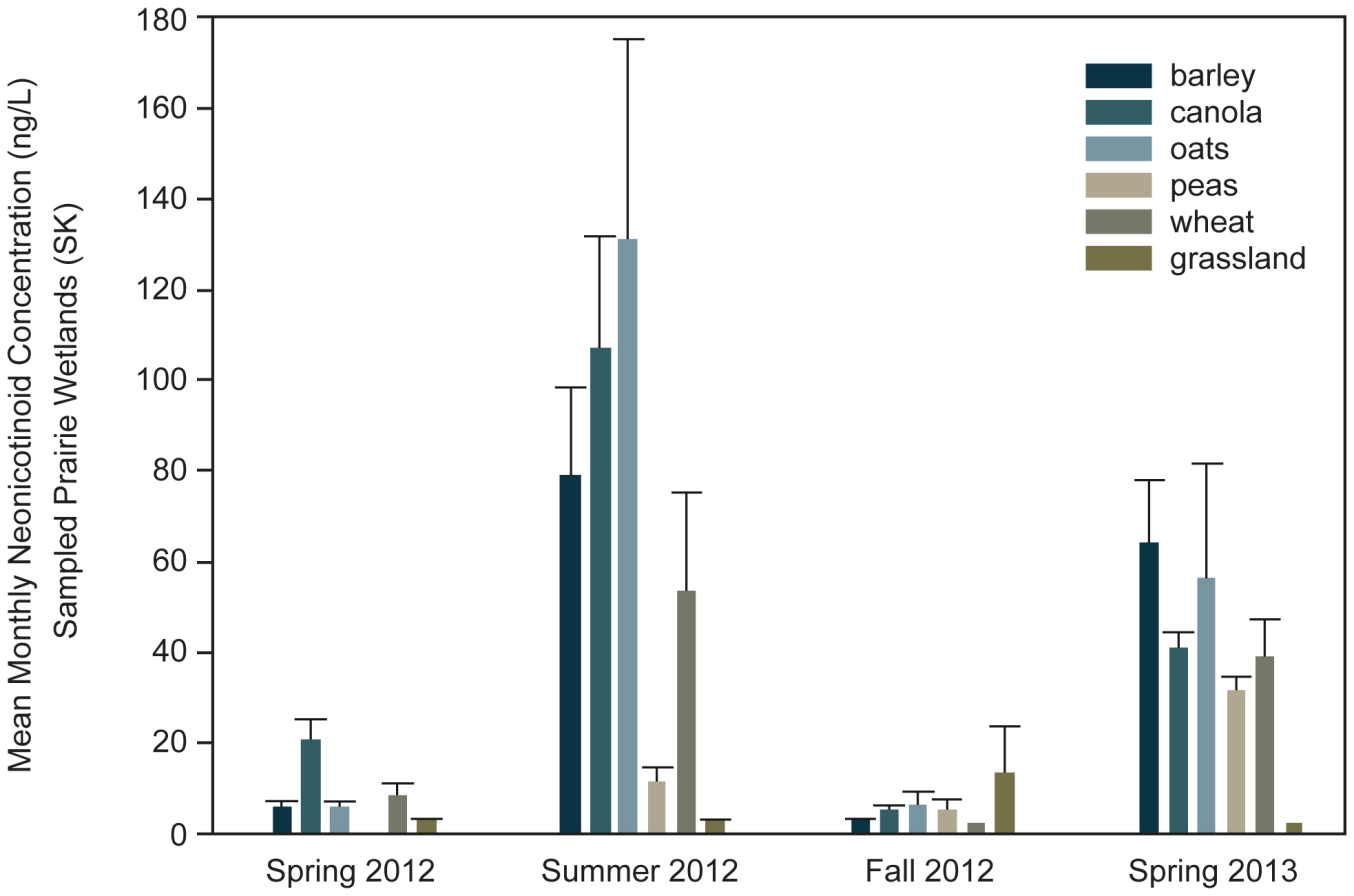
Mean total neonicotinoid water concentrations measured in wetlands sampled in central Saskatchewan over one year. Wetlands were sampled repeatedly over an annual growing cycle (spring 2012, summer 2012, fall 2012 and spring 2013). Spring 2012 wetlands reflect the 2011 crop type whereas summer 2012 and spring 2013 samples reflect new crops that were seeded in 2012.

Strong interactions between season and crop type (Table 3, Figure 4) masked the effect of season alone in the model. Declines in concentrations between summer and fall were found for barley (X^2^ = 28.01, *P* = <0.0001), canola (X^2^ = 55.13, *P* = <0.0001), and wheat (X^2^ = 11.59, *P* = 0.0007). Subsequent increases in concentrations were found the following spring 2013 for barley (X^2^ = 79.66, *P* = <0.0001), canola (X^2^ = 150.74, *P* = <0.0001), wheat (X^2^ = 53.48, *P* = <0.0001), field pea (X^2^ = 11.25, *P* = 0.0008) and oat (X^2^ = 9.75, *P* = 0.002) though not grasslands (X^2^ = 0.86, *P* = 0.35).

Because many wetlands were dry, thus not sampled in fall 2012, the significant decline and subsequent increase in neonicotinoid concentrations between seasons could result from a sampling effect rather than a within-wetland temporal trend. However, neonicotinoid concentrations were similar between ponds that dried in the fall and those that remained wet in both summer 2012 (U(df  = 1)  = 1558, *P* = 0.34) and spring 2013 (U(df = 1)  = 688, *P* = 0.99).This indicates that the observed fall decline and spring rebound in total neonicotinoid concentrations occurred within individual wetlands and was not a sampling effect.

### Neonicotinoid residues in sediment

Of the sediment samples collected during summer 2012, only 8 (6%) of the wetlands situated in fields of barley, canola, field pea and wheat contained neonicotinoid active ingredients (Table 4). The highest concentrations of each compound were thiamethoxam (max: 20.0 μg/kg, canola), imidacloprid (max: 17.5 μg/kg, canola), and clothianidin (max: 4.4 μg/kg, peas). Acetamiprid was not detected in any sediment sample.

**Table 4:** Summary of detections, arithmetic means and maximum concentrations of total neonicotinoids and active ingredients in the sediment of 134 sampled Prairie wetlands of central Saskatchewan (summer 2012).

Crop	Wetlands (n)	Detections (%)	Imidacloprid (μg/kg)		Thiamethoxam (μg/kg)		Clothianidin (μg/kg)		Acetamiprid (μg/kg)	
			Mean	Max	Mean	Max	Mean	Max	Mean	Max
Barley	18	5.6	ND	ND	ND	ND	2.6	2.6	ND	ND
Canola	61	6.6	17.5	17.5	20.0	20.0	3.4	3.9	ND	ND
Oats	3	0.0	ND	ND	ND	ND	ND	ND	ND	ND
Peas	8	12.5	ND	ND	ND	ND	4.4	4.4	ND	ND
Wheat	30	6.7	ND	ND	ND	ND	2.8	3.3	ND	ND
Grassland	14	0.0	ND	ND	ND	ND	ND	ND	ND	ND
	**134**	**6**	**17.5**	**17.5**	**20.0**	**20.0**	**3.3**	**4.4**	***	***

ND: indicates no detection of specific neonicotinoid active ingredient was found in the wetland sediment sampled.

## Discussion

To our knowledge, this is the first study that specifically assessed the scale of use of neonicotinoids in any Canadian region and level of neonicotinoid contamination in wetlands. Sales of neonicotinoid seed treatment products in Canada have rapidly expanded since the early 2000s when seed treatments using thiamethoxam (canola, mustard) and clothianidin (canola, corn) were registered. From 2002–2005, uses of thiamethoxam further increased to include seed treatment products for wheat, barley, soybean, corn, field pea, dry bean, sunflower and lentil. Globally, uses of the neonicotinoid active ingredients examined in this study have been registered for a number of foliar, soil and seed treatment applications: imidacloprid (140), acetamiprid (60), thiamethoxam (115) and clothianidin (40) [6]. The multiple seed-treatment products applied across widely distributed agricultural crops over large geographic areas presents a high degree of environmental loading and increases the potential for contamination of surface waters by neonicotinoids. According to our GIS analysis of neonicotinoid use on the Canadian Prairies, smaller areas with high application rates appear to be in regions where corn and soybean (southeastern Manitoba) and pulses or field pea (southern Saskatchewan) are extensively seeded. Mappings created by the Pesticide National Synthesis Project of “estimated agricultural use” of clothianidin, imidacloprid and thiamethoxam revealed corresponding exponential growth throughout the United States since the early 2000s. Zones of high use (presented as pounds per square mile) are similarly located in regions growing corn, soybean and crops such as cotton [35]. Our analysis also showed that large areas seeded to canola are treated with medium-high application rates. The same can be mentioned of cereals such as wheat and barley indicating neonicotinoid seed treatments are gaining popularity.

The number of previous studies in which surface waters (rivers, lakes and streams) in North America were monitored for neonicotinoids is generally limited [25]–[26], [36]–[37] with only one study on wetlands [27]. Moreover, most have focused solely on the presence of imidacloprid. For example, in California, 89% of river samples had detections with concentrations of 50 to 3290 ng/L [19]. Maximum imidacloprid values, detected in stream and agricultural run-off studies of eastern Canadian provinces (New Brunswick; Prince Edward Island), ranged from 420 ng/L to 15,880 ng/L [26], [36]–[37]. Given the physico-chemical properties of neonicotinoids, they are highly susceptible to transport into aquatic ecosystems. Neonicotinoids appear to behave similarly to other pesticides which move into aquatic systems in pulses during surface run-off and deposition of aerial spray drift [38]–[39]. It is unclear if other factors such as wind erosion of treated seeds during spring planting also influence neonicotinoid transport into wetlands. Peak concentrations of all four neonicotinoids in the water columns of wetlands in cropped fields (not grasslands) occurred in summer 2012 with a mean concentration of 91.7 ng/L, but with maximum concentrations, which frequently consisted of more than one neonicotinoid, being as high as 3110 ng/L. However, grab sampling in rivers is known to underestimate actual maxima concentrations by 1–3 orders of magnitude and average concentrations of pesticide residues by 50% [26]; the same may be true of wetlands in our study area.

Our mapping of potential neonicotinoid use within the PPR based on commonly grown crops (canola, barley, wheat, oat and field pea) suggested that wetlands situated within the PPR are exposed to neonicotinoid insecticides from seed treatments. Sampling the water column of a subset (range: fall 2012 = 80; spring 2012 = 136) of wetlands within the PPR confirmed that neonicotinoids were consistently present in 16–91% of the monitored wetlands situated in fields seeded to canola, barley, wheat, oat and field pea and in concentrations significantly higher than those detected in comparable wetlands situated in grasslands. This may have consequences for the numerous ecosystem services provided by Prairie pothole wetlands. Wetlands not only provide functions to agricultural production (e.g., clean water for livestock), they provide habitat for a large number of species such as waterbirds, amphibians and invertebrates [2]–[3]. Importantly, a small proportion of grassland wetland samples had low levels of neonicotinoids further suggesting its susceptibility to transport and potential to affect those wetlands that are isolated from agricultural production.

While maximum neonicotinoid concentrations were typically detected in wetlands situated in canola fields, wetlands in fields seeded to other crops that were monitored in the current study were also found to contain similar mean neonicotinoid levels. This may be explained by: 1) the current high economic yield of canola, resulting in frequent 2 or 3 year rotations with wheat, barley, oat or field pea [40], 2) high soil persistence that exhibits carry over between seasons and/or 3) the area of cereal crops treated by neonicotinoids has grown exponentially since 2004 leading to higher susceptibility of wetlands to neonicotinoid contamination.

Although unexpected, we found high frequency of neonicotinoid detections prior to spring planting: 36% of 136 wetlands in spring 2012 and 91% of 90 wetlands in spring 2013. Spring water samples most commonly contained clothianidin (max  = 173 ng/L) and often also contained thiamethoxam. This was despite the fact that most of the same wetlands the previous autumn had no detectable concentrations of neonicotinoids and they were not strongly retained in wetland sediments. Neonicotinoids have relatively low soil-water organic carbon partition coefficients (K_oc_) and high water solubility (e.g., clothianidin log K_oc_  = 123, solubility  = 327 mg/L) thereby limiting the potential for retention and accumulation in wetland sediments [17]. Clothianidin (DT_50_ = 148–1,155 d) and thiamethoxam (DT_50_ = 51 d) are highly persistent in soil [17] with higher reported DT_50_ values likely reflecting cold soil temperatures as frequently encountered in the Canadian Prairies. This is in agreement with regulatory studies indicating that clothianidin soil half-lives (DT_50_ values) were 385 d in Ontario, but 1386 d in North Dakota [41]. In support, a Saskatchewan study similarly found 80% of the initial (0-day) concentration in soil was still present after 775 d, indicating extremely high persistence in soils under Prairie conditions [41]. We speculate that neonicotinoid concentrations detected in Prairie wetlands in spring 2012 and 2013 were not due to persistence in water or sediment, but resulted from carryover in the soil during winter and subsequent transport to the wetlands in snowmelt runoff.

Continuous low-level contamination of wetlands by neonicotinoids both early and mid-season may have important implications for insect emergence patterns since chronic, low-level exposure may reduce invertebrate survival and growth [11], [15]. A recent study of macroinvertebrate decline in Dutch surface waters found a significant negative relationship between imidacloprid concentrations and abundance of aquatic macroinvertebrates [18]. Field studies and studies of sublethal insect toxicity from chronic exposure are generally scarce. However, the results of the current study show these compounds are continuously detected in wetlands over several months. Prolonged exposure of invertebrates to the neonicotinoids as a result of persistence, or repeated pulses to the wetlands as documented here likely lowers the dose required to cause toxicity over short-term exposure [11], [39], [42]. In addition, we detected more than one neonicotinoid in many wetlands; therefore, it is equally important to understand the cumulative effects of long-term exposure to mixtures of neonicotinoids and the potential for additive or synergistic effects of multiple neonicotinoids on aquatic organisms. Investigating single-pulse exposure of thiacloprid to stream invertebrate communities, Beketov et al. (2008) found that short-living species recovered after 10 weeks of contamination whereas long-living invertebrate species did not recover until almost 7 months later [43]. Furthermore, in the current study, peak concentrations were detected during summer months when insect emergence patterns show greatest plurality suggesting food web effects may be significant.

In Canada, an interim water quality guideline for regulation of imidacloprid for the protection of freshwater aquatic life is set at 230 ng/L [44]. Other guidelines for imidacloprid have been set by the U.S. EPA at 1050 ng/L for long term exposure and 35,000 ng/L for acute pulse events [45]. The European Water Framework Directive applies a Maximum Permissible Concentration (MPC) of 65 ng/L for long term exposure or Maximum Acceptable Concentration (MAC) of 200 ng/L for acute exposure. In regard to thiamethoxam, the US EPA has a published guideline for acute exposure set at 17,500 ng/L. Recently, Mineau and Palmer (2013) recommended 10–30 ng/L as a protective concentration under long term exposure based on a species sensitivity distribution analysis and the HC_5_ using available chronic toxicity studies [46]. The mean and maximum concentrations of clothianidin and thiamethoxam detected in this study frequently exceeded many of these guidelines based on the chemically related compound, imidacloprid. For example, clothianidin was detected at concentrations up to 14 times above the modest Canadian benchmark for imidacloprid. However, this must be interpreted cautiously because there are currently no accepted aquatic benchmarks for either of clothianidin or thiamethoxam in Canada and most international regulatory agencies are currently reviewing their existing guidelines.

## Conclusion

Modelling neonicotinoid seed treatment applications within the PPR in Canada revealed increasing use over a large geographic area. Due to the intensity of crop rotations with neonicotinoid treated crops and the high environmental persistence of neonicotinoids in soil, the potential for environmental loading and transport into wetlands appears high. Monitoring the water column of a subset of wetlands within the PPR in Saskatchewan confirmed that neonicotinoid insecticides were repeatedly present in many of the wetlands sampled. Our findings have important implications for wetland ecosystem services such as litter breakdown, nutrient cycling and aquatic insect production, with potential consequences for wetland dependent species (e.g., amphibians, waterfowl; aerial insectivorous birds). In order to fully understand the effects of neonicotinoids on PPR wetlands, we recommend future studies: 1) determine levels of neonicotinoid contamination in other regional aquatic systems and across a landscape level scale; 2) determine the ecological features that make PPR wetlands susceptible to neonicotinoid contamination; and 3) identify insect abundance, productivity and emergence responses to chronic and repeated neonicotinoid exposures.

## Supporting Information

Figure S1 **Map of modelled distribution of neonicotinoid use across Prairie Canada: Alberta, Saskatchewan and Manitoba (2011).**
(TIF)

Figure S2 **Map of modelled distribution of neonicotinoid use across Prairie Canada: Alberta, Saskatchewan and Manitoba (2010).** Remote sensing crop data for Manitoba was unavailable from Agriculture and Agri-Food Canada in 2010.
(TIF)

Figure S3 **Map of modelled distribution of neonicotinoid use across Prairie Canada: Alberta, Saskatchewan and Manitoba (2009).**
(TIF)

Table S1 **Details of MS/MS transitions and operational parameters for neonicotinoid analysis.**
(DOCX)
