# Correction: Widespread Use and Frequent Detection of Neonicotinoid Insecticides in Wetlands of Canada's Prairie Pothole Region

**DOI:** 10.1371/journal.pone.0101400

**Published:** 2014-06-24

**Authors:** The PLOS ONE Staff

There is an error in the third to last sentence of the Abstract. The neonicotinoid concentration units should be listed as µg/kg rather than ng/L. The correct sentence is:

“Sediment samples collected during the same period rarely (6%) contained neonicotinoid concentrations (which did not exceed 20 µg/kg).”
