# Supplementary figures and images for: Widespread Use and Frequent Detection of Neonicotinoid Insecticides in Wetlands of Canada's Prairie Pothole Region
Source: PLoS One. 2014 Mar 26;9(3):e92821. doi: 10.1371/journal.pone.0092821 (PMC3966823; doi:10.1371/journal.pone.0092821)

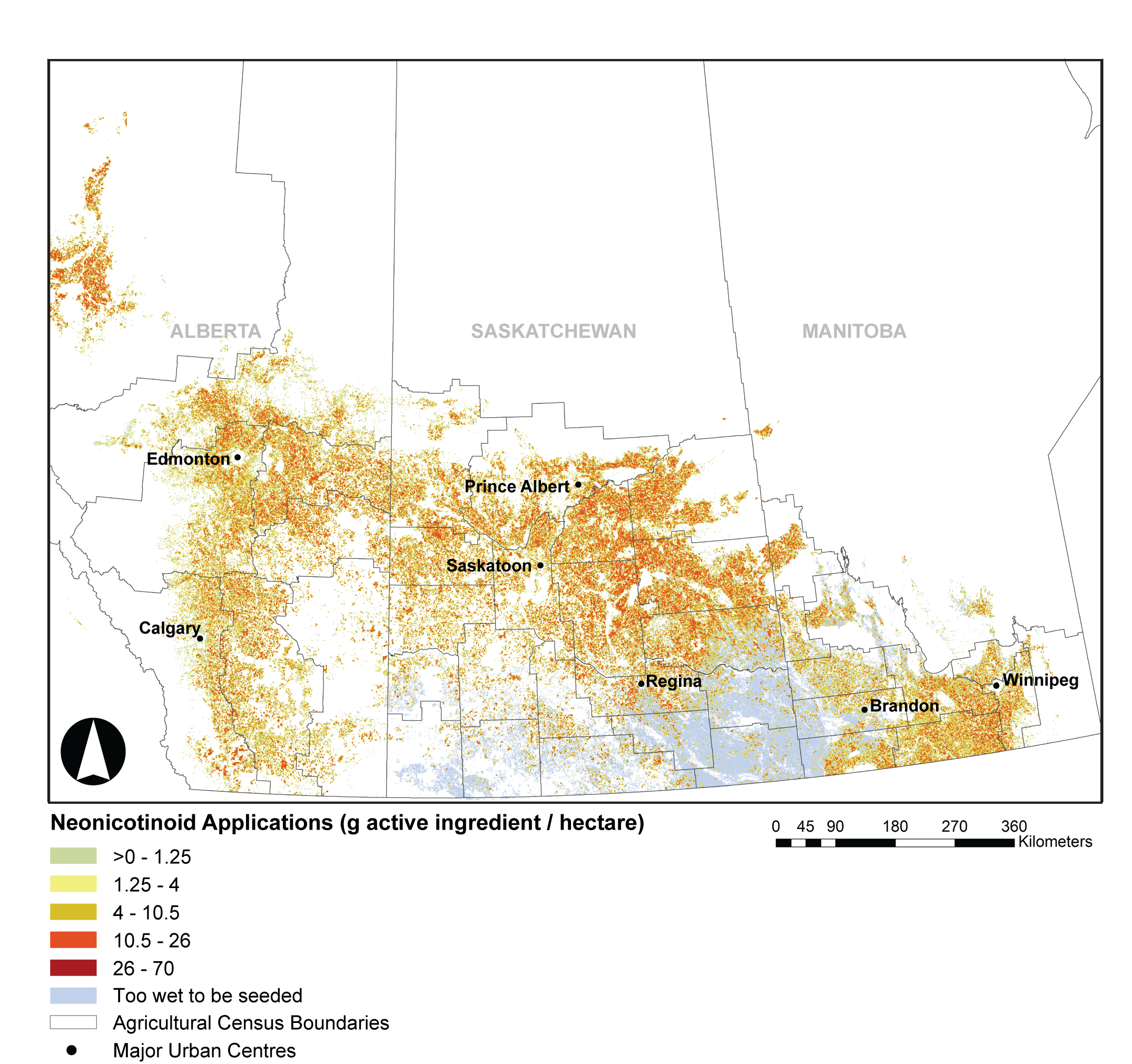

Supplement: Figure S1 — Map of modelled distribution of neonicotinoid use across Prairie Canada: Alberta, Saskatchewan and Manitoba (2011). (TIF) [file pone.0092821.s001.tif]

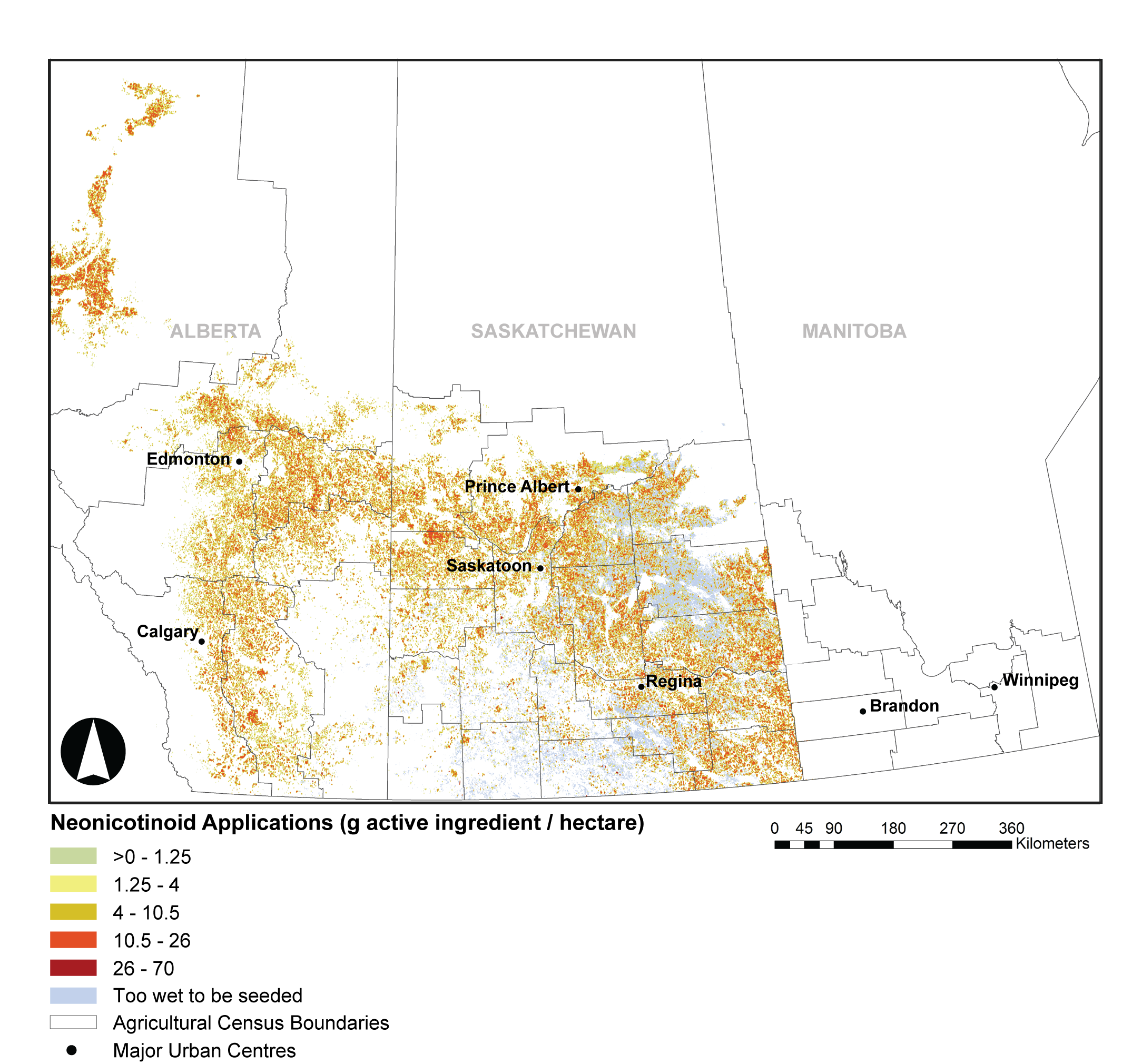

Supplement: Figure S2 — Map of modelled distribution of neonicotinoid use across Prairie Canada: Alberta, Saskatchewan and Manitoba (2010). Remote sensing crop data for Manitoba was unavailable from Agriculture and Agri-Food Canada in 2010. (TIF) [file pone.0092821.s002.tif]

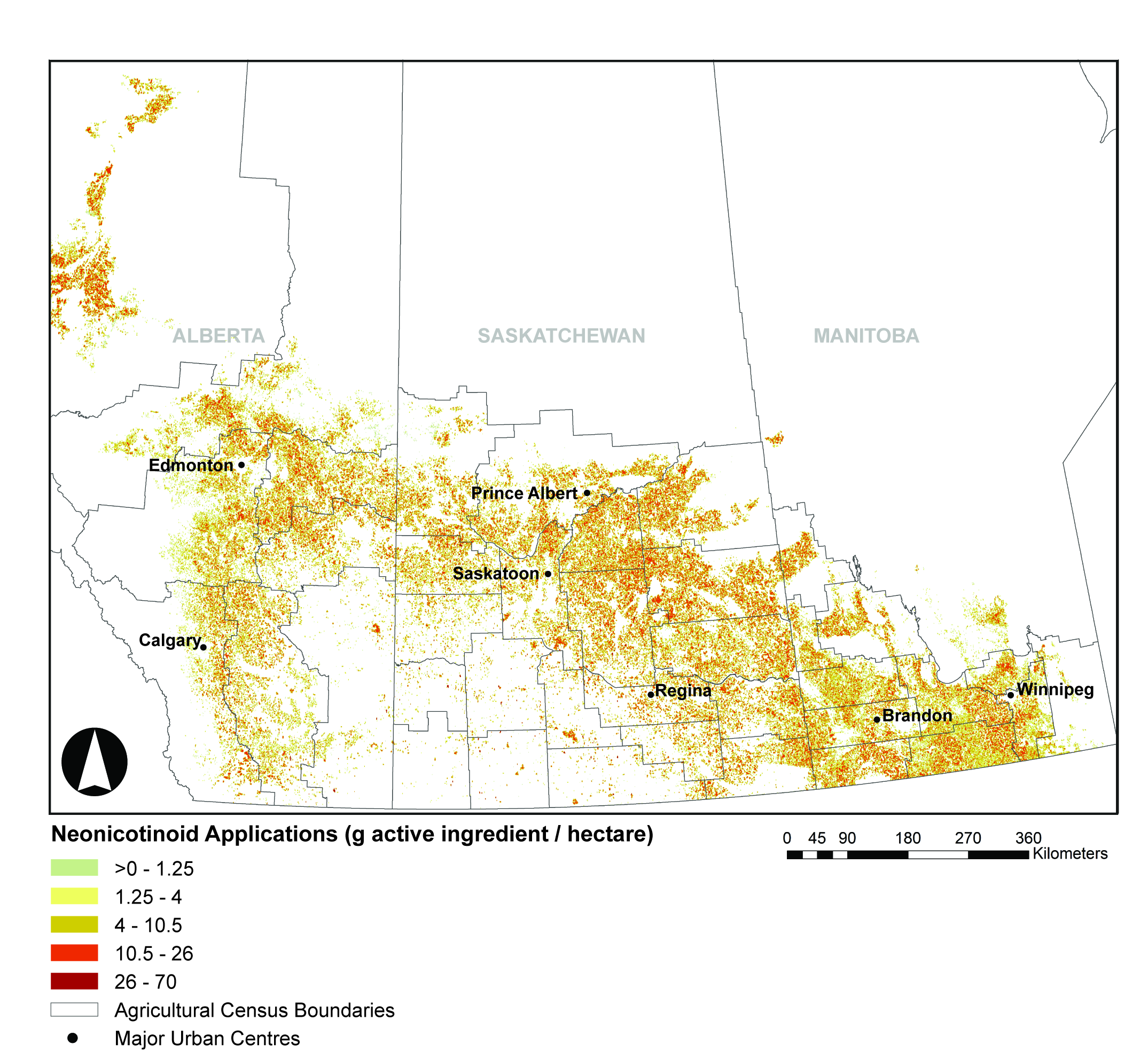

Supplement: Figure S3 — Map of modelled distribution of neonicotinoid use across Prairie Canada: Alberta, Saskatchewan and Manitoba (2009). (TIF) [file pone.0092821.s003.tif]
