# Supplementary material for: Widespread Use and Frequent Detection of Neonicotinoid Insecticides in Wetlands of Canada's Prairie Pothole Region
Source: PLoS One. 2014 Mar 26;9(3):e92821. doi: 10.1371/journal.pone.0092821 (PMC3966823; doi:10.1371/journal.pone.0092821)
Supplement: Table S1 — Details of MS/MS transitions and operational parameters for neonicotinoid analysis. (DOCX) [file pone.0092821.s004.docx]

| Neonicotinoid Insecticides | MRM Transition Monitored (m/z) | Cone Voltage (V) | Collision Energy (eV) | Retention Time (min) |
| --- | --- | --- | --- | --- |
| Thiamethoxam | 292.1 > 211.0 292.1 > 181.0 | 27 27 | 18 18 | 3.60 3.60 |
| Clothianidin | 250.1 > 169.0 250.1 > 131.9 | 19 19 | 18 18 | 4.76 4.72 |
| Imidacloprid | 256.0 > 209.3 256.0 > 175.2 | 27 27 | 18 18 | 5.24 5.21 |
| d_4_-Imidacloprid | 260.1 > 213.1 260.1 > 179.2 | 27 27 | 18 18 | 5.22 5.25 |
| Acetamiprid | 223.1 > 126.1 223.1 > 56.0 | 27 27 | 18 18 | 6.79 6.82 |
